# Pregnancy Intention and Pregnancy Outcome: Systematic Review and Meta-Analysis

**DOI:** 10.1007/s10995-016-2237-0

**Published:** 2017-01-16

**Authors:** Jennifer A. Hall, Lorna Benton, Andrew Copas, Judith Stephenson

**Affiliations:** 1 Research Department of Reproductive Health, UCL Institute for Women’s Health, London, UK; 2 Department of Population Policy and Practice, UCL Institute of Child Health, London, UK; 3 Department of Infection and Population Health, UCL Institute of Epidemiology and Health Care, London, UK

**Keywords:** Systematic review, Meta-analysis, Pregnancy intention, Miscarriage, Stillbirth, Low birthweight, Neonatal mortality

## Abstract

*Introduction* Previous systematic reviews concluded that rigorous research on the relationships between pregnancy intentions and pregnancy outcomes is limited. They further noted that most studies were conducted in high-income countries and had methodological limitations. We aim to assess the current evidence base for the relationship between pregnancy intention and miscarriage, stillbirth, low birthweight (LBW) and neonatal mortality. In March 2015 Embase, PubMed, Scopus and PsychInfo were searched for studies investigating the relationship between pregnancy intention and the outcomes of interest. *Methods* Studies published since 1975 and in English, French or Spanish were included. Two reviewers screened titles and abstracts, read the full text of identified articles and extracted data. Meta-analyses were conducted where possible. *Results* Thirty-seven studies assessing the relationships between pregnancy intention and LBW were identified. A meta-analysis of 17 of these studies found that unintended pregnancies are associated with 1.41 times greater odds of having a LBW baby (95%CI 1.31, 1.51). Eight studies looking at miscarriage, stillbirth or neonatal death were found. The limited data concerning pregnancy loss and neonatal mortality precluded meta-analysis but suggest these outcomes may be more common in unintended pregnancies. * Discussion* While there seems to be an increased risk of adverse pregnancy outcome in unintended pregnancies, there has been little improvement in either the quantity of evidence from low-income countries or in the quality of evidence generally. Longitudinal studies of pregnancy intention and pregnancy outcome, where pregnancy intention is assessed prospectively with a validated measure and where analyses include confounding or mediating factors, are required in both high- and low-income countries.

## Significance

*What is already known on this subject?* Pregnancy intention may be associated with adverse pregnancy outcomes. Previous systematic reviews concluded that insufficient attention has been paid to investigating these relationships. They further noted that most studies were conducted in high-income countries and had methodological limitations that could invalidate their findings.

*What this study adds?* This review provides an updated meta-analysis of the relationship between pregnancy intention and LBW. In addition persistent gaps and flaws in the literature are demonstrated. Retrospective, cross-sectional studies predominate, despite their limitations. Longitudinal studies with analyses that include confounding or mediating factors are required in both high- and low-income countries.

## Background

It may seem self-evident that unintended pregnancies would be associated with adverse outcomes. However, the evidence base for the relationships between pregnancy intention and maternal and neonatal outcomes is mixed. Between 2008 and 2011 there were three systematic reviews published on this topic (Gipson et al. 2008; Shah et al. 2011; Tsui et al. 2010). These reviews concluded that scant attention had been paid to investigating the relationships between pregnancy intention, health behaviours and maternal and child health outcomes, that the existing research was ‘older and methodologically limited’ (p157) (Tsui et al. 2010) and that there are *‘*persistent gaps in the literature, indicating a need for more studies in developing countries’ (p18) (Gipson et al. 2008).

Only Shah et al. (2011) conducted a meta-analysis, calculating a crude odds ratio (OR) for unintended pregnancies of 1.36 (95% confidence interval (95%CI) 1.25, 1.48) for low birthweight (LBW) and 1.31 (95%CI 1.09, 1.58) for preterm birth (PTB). The meta-analysis was conducted on unadjusted estimates, given the variation in confounders adjusted for by different studies, and may therefore overestimate the relationship. Almost all studies were conducted in Europe or the USA meaning these findings may not have relevance to low-income countries (LICs). Moreover most studies were retrospective, cross-sectional surveys using a single question to dichotomise pregnancies into intended and unintended. These are the methodological limitations referred to by Tsui et al. (2010) because they over-simplify the complex construct of pregnancy intention, resulting in misclassification bias, and introduce recall bias given the time elapsed between the pregnancy and the timing of assessment (up to 5 years after birth). The temporal separation between pregnancy intention and outcome is lost making any assessment of mechanism of effect, or cause and effect, impossible.

Five years on this systematic review aims to assess the current evidence base for the relationships between pregnancy intention and adverse pregnancy outcomes. In addition to LBW we review the evidence for miscarriage, stillbirth and neonatal mortality, conducting meta-analyses where possible and comparing findings for LICs and high-income countries (HICs).

## Methodology

### Search Strategy and Keywords

The literature review and meta-analyses were conducted in line with the ‘Meta-analysis of Observational Studies in Epidemiology (MOOSE)’ guidelines (Stroup et al. 2000). Searches were carried out on the electronic databases Embase, PubMed and Scopus in March 2015. Where possible Medical Subject Headings were used. For unintended pregnancy, the exposure, stems and words covering the concepts of pregnancy, fertility, birth, child, intention, want, planning or timing were used and were combined using the Boolean operator ‘or’.

Definitions of the outcomes are shown in Box 1. For the outcomes, full and truncated terms, acronyms e.g. LBW for low birthweight, synonyms such as neonatal death and neonatal mortality, and the generic ‘pregnancy outcome’ were combined with ‘or’. The results of the separate pregnancy intention and outcome searches were then combined with ‘and’. The search also included the outcome of postnatal depression and was additionally conducted on PsychInfo. The findings of the relationship between pregnancy intention and postnatal depression are presented elsewhere.

Box 1 Definitions of outcomes of interest

**Table:** 

Miscarriage	A pregnancy lost before 28 weeks’ gestation
Stillbirth	A baby born with no signs of life at or after 28 weeks’ gestation
Low birthweight	A baby born weighing <2500 g regardless of gestation
Neonatal death	A baby born alive but who dies within the first 28 days of life

### Inclusion and Exclusion Criteria

Observational studies of any design that investigated the relationship between pregnancy intention and at least one of the outcomes of interest were eligible for inclusion in the review. Studies in restricted populations, such as teenagers or those with particular medical conditions, were excluded, as these were not representative of the general population. Sufficient information on how pregnancy intention was assessed and reported had to be provided, but no restrictions were placed on the timing or method of the assessment. Articles published since 1975 and in English, French or Spanish were eligible for inclusion.

JH and LB reviewed the titles and abstracts independently. All abstracts selected by either reviewer were retained for full-text review. The references of these articles were also reviewed to identify any additional eligible studies.

### Quality of Study and Risk of Bias

The potential sources of bias by which the studies were assessed included how the sample was selected, whether the sample was representative, sample size, how the exposure and outcomes were measured (whether they were validated measures and the timing of the assessment), confounders that were controlled for, loss to follow-up and the type of analysis conducted. Where appropriate funnel plots were created to investigate publication bias and/or small study effects.

### Data Extraction

Data were extracted from the studies independently by JH and LB, using a template designed for this review, and differences resolved by discussion. Data extracted included the location, population, measure and timing of pregnancy intention, proportion of pregnancies classed as unintended (and mistimed or ambivalent if presented), method of assessing the outcome, outcome data and confounders controlled for.

### Meta-Analysis

Where there were sufficient studies with data available for the primary outcomes, raw data were extracted from the papers and meta-analyses conducted in Stata to calculate an overall effect size estimate (odds ratio) for the studies. Heterogeneity between studies was assessed before deciding whether to conduct a fixed-effects or random-effects analysis. Given the expected variety of study populations, study design and assessment method it was decided *a priori* to stratify the analysis by location (using World Bank country classifications) and whether pregnancy intention was assessed during pregnancy or afterwards.

## Results

### Search Results

The four database searches looking at all outcomes returned a total of 3159 hits combined. 945 of these were duplicates, 40 were excluded as they were pre-1975 and 117 on the basis of language. Following review of the title and abstract 1973 were removed, mostly because they were not addressing the relationship between pregnancy intention and an outcome of interest.

**Fig. 1:**
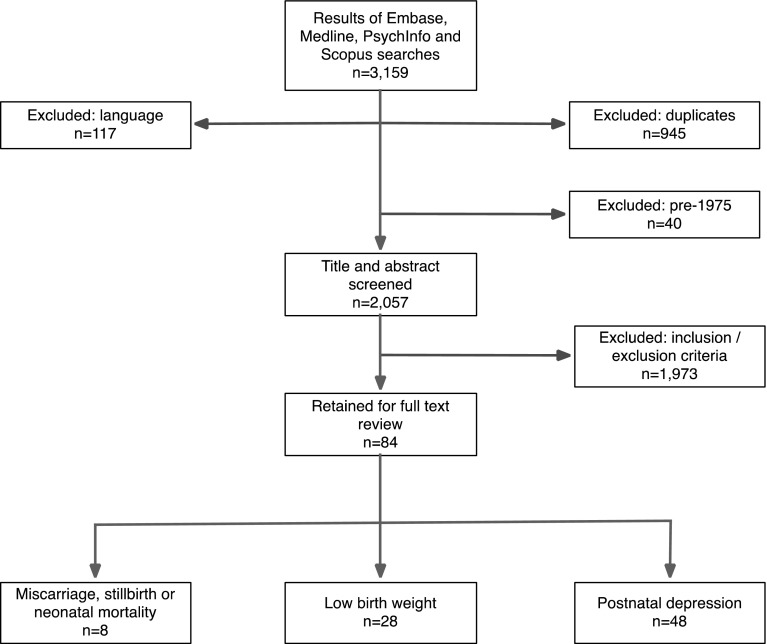
Flow chart of selection of studies for the literature review

There were 84 studies relevant to the primary outcomes: eight to miscarriage, stillbirth or neonatal death, 28 to low birthweight and 48 to postnatal depression. The flowchart for the literature review is shown in Fig. 1.

### Pregnancy Intention and Miscarriage, Stillbirth or Neonatal Death

Eight studies that addressed the relationships between pregnancy intention and miscarriage, stillbirth or neonatal death were identified from the literature review. One was excluded on full text review as it contained no data and an additional study was identified from the references of other papers, giving eight studies in total. The characteristics of these studies are included in Table 1.

**Table 1:** Characteristics of studies investigating the relationships between pregnancy intention and miscarriage, stillbirth or neonatal mortality

First author, year, location	Study population (size)	Methodology	Measure of intention and timing of assessment	Proportion of UIPs	Result for miscarriage	Result for still-birth	Result for neonatal death	Result for post-neonatal mortality	Result for infant mortality	Result for child mortality	Limitations/comments
Dawen et al. (2014). Data collected: unknown. UK (HIC)	Women presenting to an early pregnancy unit in London over 3 years (4139)	Retrospective observational cohort study of pregnant women who had documentation of outcome and whether the pregnancy was planned or unplanned	Question not included. Antenatal	35.9%	No difference (40.5% versus 42.5%)						Limited information as only an abstract, no raw data so can’t calculate OR; not even clear which way round the percentages are
Wellings et al. (2013). Data collected: 2010-12. UK (HIC)	A nationally representative sample of 15 162 men and women aged 16–74 years, looked at women aged 15–44 who had a pregnancy in the last year (5686)	Cross-sectional survey	London Measure of Unplanned Pregnancy. <12 months postnatally	16.2% unplanned 29% ambivalent	No significant difference on univariate analysis (unplanned 33.6%, planned 35.3%)						Used LMUP but dichotomised and did so at unusual cut-point. Retrospective, cross-sectional data. No adjusted analysis presented
Singh and Mahapatra (2013). Data collected: 1998–2003. India (LMIC)	Currently married women aged in 15–39 in four states in 2002–03 who had been interviewed in the National Family Health Survey in 1998–99 were re-interviewed (2108)	Surveys completed at the two time points and asking about the time in between	Fertility intentions in non-pregnant women: Would you like to have (a/nother) child or would you prefer not to have any more children? How long would you like to wait from now before the birth of (a/nother) child? For pregnant women: DHS question. Pre-conception	49.0%			Adjusted OR 1.83 (1.01, 3.34)		aOR 1.52 (0.95, 2.45)		Only currently married women, limited age range. Asked about future fertility intentions and used this to assign wantedness to future pregnancies, without recognising that this could have changed. Excluded women who were ‘unsure’. Pregnancies that did not result in a live birth were not included
Assefa et al. (2012). Data collected: 2009–10. Ethiopia (LIC)	Women who became pregnant in one of 12 kebeles during the study period	Women visited every 3 months and tested for pregnancy, then visited monthly to determine pregnancy outcome	Question not included. Antenatal	31.8%	Crude HR 3.0 (2.15, 4.15), adjusted HR 2.2 (1.56, 3.11)						Will have missed some early miscarriages, but this should be minimal. Can’t differentiate between miscarriage, abortion and stillbirth so all combined into pregnancy loss
Singh et al. (2012). Data collected: 2005–06. India (LMIC)	A nationally representative sample of 124 385 women aged 15–49 (51,555)	Cross-sectional survey	DHS question, <12 months postnatally	20.3%			aOR mistimed 1.82 (1.16, 2.84), aOR unwanted 2.22 (1.17, 4.24)	aOR mistimed 2.60 (1.07, 6.76), aOR unwanted 3.64 (1.39, 9.51)		aOR mistimed 1.37 (0.48, 3.89), aOR unwanted 5.92 (1.48, 23.7)	Retrospective DHS style questions, up to 5 years after the birth. Only live births included. ‘Child’ mortality actually 12–35 months
Chalasani et al. (2007). Data collected: 1982–2002. Bangladesh (LIC)	Women in the Sample Registration System in the Jessore and Sirajgonj districts (3283)	Baseline and quarterly surveillance from 1982 to 2002	Women were asked prospective preferences about how many further children they wanted of each sex; subsequent births were then categorised as wanted, “up to God”, and unwanted. Pre-conception	41.0%			Adjusted OR 2.09 (p < 0.001)	Adjusted OR 2.00 (p < 0.001)		Deaths from 12 to 60 months. Adjusted OR 1.38	
Bustan and coker (1994). Data collected: 1959–1966. USA (HIC)	Pregnancies in women who were members of the Kaiser Health Plan in the East Bay Area of San Francisco (20,754)	Women recruited on confirmation of pregnancy, interviewed and data extracted from medical records	Wording of question not included but asked about the attitude of the woman and her partner to the pregnancy. Antenatal	14.4%	Crude RR 1.4 (1.0, 1.8), adjusted RR 1.1 (0.9,1.5)		Crude RR 2.7 (1.8, 4.3), adjusted RR 2.4 (1.5, 4.0)	Crude RR 0.8 (0.4, 1.9), adjusted RR 0.9 (0.5,1.9)			Excluded unmarried women and those who said that their attitude to the pregnancy was different to their husbands. Also women who had access to this health care plan—suggests low-risk population raising issues of representativeness
Laukaran and Van Den Berg (1980). Data collected: 1958–67. USA (HIC)	Women enrolled in the child health and development studies who were a member of the Kaiser Foundation Health Plan in the San Francisco East Bay Area (12391)	Utilises data from the Child Health and Development studies. Ever-married women receiving antenatal care at KFH. Outcome data from medical records	How do you feel about having a baby now?’ Antenatal	6.2% strong negative attitude		The relative risk (RR) adjusted for parity and husband’s occupation was 1.80 (p = 0.003) and adjusted for mother’s age and parity was 1.78 (p = 0.002)					Prospective study but measure of intention not comparable. Analysis only conducted on ever-married white women and only controlled for a couple of confounders

### Pregnancy Intention and Miscarriage or Stillbirth

There are very few data on the relationship between pregnancy intention and miscarriage or stillbirth; just two studies in a HIC and one in a LIC. Dawen et al. found no relationship between unintended pregnancy and miscarriage in women attending an early pregnancy unit in London, UK (Dawen et al. 2014). Using the London Measure of Unplanned Pregnancy (LMUP) in the UK, Wellings et al. (2013) noted that unplanned pregnancies were more likely to end in abortion, but there was no difference in the proportion ending in miscarriage.

A study in Ethiopia identified pregnant women in the community, assessed their pregnancy intention and followed them up monthly until the outcome of the pregnancy was known (Assefa et al. 2012). Miscarriage, induced abortion and stillbirth were analysed as a composite of ‘pregnancy loss’. Using a robust, prospective methodology they found an adjusted hazard ratio for pregnancy loss of 2.2 (95%CI 1.56, 3.11) for unintended compared to intended pregnancies.

### Pregnancy Intention and Neonatal Mortality

There have been more studies looking at neonatal mortality in both HICs and LICs. Two studies in the USA found that unintended pregnancies had a greater risk of neonatal mortality. In California, Laukaran and van den Berg (1980) found a relative risk (RR) of perinatal mortality of 1.80 (p = 0.003) (adjusted for parity and husband’s occupation).^1^ Bustan and Coker (1994) found an adjusted RR of 2.4 (95%CI 1.5, 4.0) for neonatal mortality in married women with health insurance who received early antenatal care but who felt negative about their pregnancies during pregnancy. The fact that an increased risk of mortality was found in these two low-risk populations is noteworthy. However, these studies are both old (data were collected in the 1950s and 60s) and their current applicability may be limited given changes in mortality rates and the availability of abortion since these data were collected.

Three studies in LICs looked at neonatal mortality; two from India (Singh et al. 2012; Singh and Mahapatra 2013) and one from Bangladesh (Chalansani et al. 2007). All three studies found increased risk of neonatal mortality, as shown Table 2. Singh and Mahapatra (2013) and Chalansani et al. (2007) both used prospective fertility intentions and used siblings to control for unobserved heterogeneity at the level of the family, making these studies particularly robust.

**Table 2:** Adjusted odds ratios and 95%CI for pregnancy intention and neonatal mortality

Study	Neonatal mortality	Post-neonatal mortality	Deaths from 12 to 35 months
Singh et al. (2012), India			
Mistimed births	1.82 (1.16, 2.84)	2.06 (1.07, 6.76)	1.37 (0.48, 3.89)
Unwanted births	2.22 (1.17, 4.24)	3.64 (1.39, 9.51)	5.92 (1.48, 23.7)
Chalasani et al. (2007)			
Unwanted births	2.09 (p < 0.001)	2.00 (p < 0.001)	–

	Neonatal mortality	Infant mortality	
Singh and Mahapatra (2013)			
Unwanted births	1.83 (1.01, 3.34)	1.52 (0.95, 2.45)	–

### Pregnancy Intention and Low Birthweight

The search identified 28 studies potentially relating to pregnancy intention and low birthweight after title and abstract screening; a further nine were added from reference searches. The characteristics of these 37 studies are shown in Table 3.

**Table 3:** Characteristics of studies investigating the relationships between pregnancy intention and low birthweight

Author, year, location	Study population (size)	Methodology	Measure of intention and timing of assessment	Proportion of UIPs	Measure of birth weight	Confounders controlled for	Result for LBW, unadjusted	Result for LBW, adjusted	Limitations/comments	In meta-analysis
Kost and Lindberg (2015), USA	Non-multiple live births in 3 years preceding the interview (4184)	Analysis of pooled NSFG data from 2 survey rounds	NSFG question plus How much later did you want to become pregnant? And a multi-dimensional measure assessing trying, wanting and happiness. Postnatal mixed	30%	LBW <2500	Age, SES, marital status, education, parity, ethnicity, health insurance	6% intended, 12% UIP, p < 0.05	Not able to calculate /compare results presented	Retrospective, recall, live births only, but large, representative survey. Strong analytic methodology	Yes
Wado et al. (2014), Ethiopia	Community-based cohort of pregnant women in 11 kebeles in south-western Ethiopia (537)	Interview during pregnancy with follow up within 72 h to of birth to weigh the baby	DHS question. Plus questions about happiness with pregnancy. Antenatal	41%	LBW <2500	Age, education, SES, MUAC, AND, social support	OR 2.31 (1.25, 4.27)	RR 1.25 (0.73, 2.14) for mistimed, RR 2.08 (1.02, 4.23) for unwanted	Excluded stillbirths and neonatal deaths	Yes
Lindberg et al. (2014), USA	Women who delivered live births in Oklahoma between 2004 and 8 and completed a follow-up survey in 2006–10 (8327)	Analysis of PRAMS data (self-administered cross-sectional survey) for Oklahoma linked to 2 years follow up survey	PRAMS question plus How much later did you want to become pregnant? Postnatal 1–6 months	49%	LBW <2500	Age, SES, marital status, education, parity, ethnicity, smoking/alcohol, health insurance	8% in UIP, 7% in IP, p < 0.05	OR 1.19 (0.93, 1.53) unwanted, 0.85 (0.70, 1.02) for mistimed > 2years, 0.95 (0.80, 1.12) mistimed < 2 years	Retrospective, recall, live births only, but large, representative survey	Yes
Kayode et al. (2014), Ghana	Women in 2003/2008 DHS who had given birth (5013)	Analysis of DHS data	DHS questions. Postnatal mixed	30.6%	LBW <2500	None	18% in UIP, 16% in IP, no sig test	–	DHS style questions and methods, analysis is focused on LBW so no data on confounding of UIP	Yes
Saedi et al. (2013), Iran	Unclear	Unclear	Unclear	28%	LBW <2500	Not clear	8.5% in unwanted, 11% in wanted, not sig	–	Too little information on methodology, poor quality	No
McCrory and McNally (2013), Ireland	Children aged 9 months between Sept 2008-April 2009 (11,134)	Weighted sample from Child Benefit Register, 64.5% response rate, about 25% of all births. Families interviewed in person and CASI at 9 months	Wording not clear but ‘asked the mother whether she intended to become pregnant before the study child was conceived’. Postnatal 7–12 months	41%	LBW <2500	Age, SES, marital status, education, parity, ethnicity, smoking, ANC	OR 1.11 (0.94, 1.31)	RR 1.01 (0.83, 1.22)	Retrospective, recall, live births only, but large, nationally representative survey. Mixed up categories of intention	Yes
Flower et al. (2013), UK	Singleton babies born in UK in 2000–01 (18,178)	Analysis of Millennium Cohort Study data	Were you planning to get pregnant or was it a surprise? Postnatal 7–12 months	43%	LBW <2500	Age, SES, marital status	5.2% in planned, 7.2% in UIP	OR 1.24 (1.04, 1.48)	Adjusted 1.24. Retrospective, recall, live births and singletons only, but large, nationally representative survey	Yes
Flores et al. (2010), USA	Utah women delivering single live birth >20 weeks gestation (190,948)	Analysis of PRAMS data for Utah, linked to birth certificates	PRAMS question. Postnatal 1–6 months	39%	LBW <2500	None	In some ethnic groups, women with UIP were significantly more likely to have a LBW	–	Abstract only so can’t make a thorough assessment but using PRAMS data, which from other papers appears to be good. Most comparisons are between different ethnicities	No
Postlethwaite et al. (2010), USA	Women insured with Kaiser Permanante (1671)	Retrospective medical record review of women receiving their first ANC visit at one of the KP obstetric offices in 2002, random sample of 400 taken from each clinic, 2400 records reviewed	At the time that you conceived, did you want to become pregnant (intended vs. unintended), did you want to become pregnant but not at this time (mistimed), or did you not want to become pregnant at all (unwanted)? Antenatal	37.8%	SFGA	None	4.8% unwanted, 2.3% intended, says not sig	–	All women had a KP pre-paid health plan and access to ANC. They had earlier initiation of ANC than nationally (88–89% v 84%), lower levels on UIP (37.8% v 49% p < 0.0001) and low levels of SFGA (3.37%)—suggests that this is an advantaged population. SFGA only, data not comparable	No
Iranfar et al. (2009), Iran	Women on the postnatal ward at Kermanshah maternity hospital (114)	Case-control study on postnatal ward	Questions on birth control method and stopping time, planning for pregnancy and tendency to abortion. Postnatal <1 month	47%	BW <3000 g and average birthweight	None	35.7% in UIP, 27% in IP, p = 0.51	–	Cases should have been those with low birthweight, not those with UIP. Measure of intention not clear - seems to be that it was assumed to be UIP if the woman had been using contraception. Presents <3000 g so not comparable data	No
Hohmann-Marriott (2009), USA	Participants of the Early Childhood Longitudinal Study (ECLS) birth cohort where biological father resident at 9 months (5788)	Analysis of couple level data from interviews at 9 months in the ECLS on conception, pregnancy and birth, linked with birth certificate data	At the time that you/your partner became pregnant with your baby, did you yourself actually want to have a(nother) baby at some time? Did you/your partner become pregnant sooner than you wanted, later than you wanted to at about the right time? Postnatal 7–12 months	Can’t do	LBW <2500		OR 1.36, no 95%CI given, says not sig	No data	Inclusion criteria of father resident at 9 months will bias the results to the null because fathers of UIPs are less likely to be living with the child at 9 months than fathers of IP. Data presented on couple intentions is incorrect. Can’t extract numbers to calculate ORs. Presented OR is mother only - not comparable - would need TO be combination of mother only and both v father only and neither but data not available	No
Shaheen et al. (2007), Egypt	Ever married women in the Demographic and Health Survey (DHS) (2379)	Analysis of DHS data	DHS question. Postnatal 7–12 months	18%	Women’s assessment of size	Age Parity	UIP more likely to report smaller than average size OR 1.34 (1.02, 1.76)	–	Outcome assessed by women’s report of size - subjective. Only married women and only live births therefore underestimates UIP rate	No
Mohllajee et al. (2007), USA	Women who gave birth in 18 states in 1996–99, multiple births excluded (87,087)	Analysis of PRAMS data for 18 states linked to birth certificates	PRAMS questions. Postnatal 1–6 months	47%	LBW <2500 and LBW <10%	Age Parity Marital status Education Smoking / alcohol ANC Previous LBW / PTB	6.8% UIP, 5% IP, p < 0.01	OR 1.06 (0.97, 1.16) unwanted, OR 0.92 (0.86, 0.97) mistimed, OR 1.15 (1.02, 1.29) ambivalent	Retrospective, recall, live births only	Yes
Collier and Hogue (2007) USA	Women who gave birth in Georgia in 1996–97 (211,716)	Analysis of PRAMS data for Georgia linked to birth certificates	PRAMS questions. Postnatal 1–6 months	63%	LBW <2500	None	RR 1.37 (1.08, 1.72)	–	Retrospective, recall, live births only	Yes
Rafati et al. (2005), Iran	Births in two hospitals in Tehran (460)	Case-control study on postnatal ward—cases = LBW, controls = 2 babies >2500 g born consecutively after each case. Neonates with complications excluded	Unclear. Postnatal <1 month	3%	LBW <2500 and average birthweight	None	In LBW babies 6% were UIP, in normal birthweight 1% were UIP, p < 0.001	–	Measure of intention not described	No
D’Angelo et al. (2004), USA	15 states (25,027)	Analysis of PRAMS data for 15 states linked to birth certificates	PRAMS questions. Postnatal 1–6 months	43%	LBW <2500	None	9.6% in unwanted, 6.5% in wanted, p < 0.001	–	Retrospective, recall, live births only	Yes
Durousseau and Chavez (2003), USA	California, term births to >15 yo during February to May 1999 and from February to May 2000 Inc. twins and triplets (5941)	Uses California’s Maternal Infant Health Assessment (MIHA) an annual population-based self-administered mail survey with telephone follow up for non-responders that collects information about pregnancy-related conditions and behaviours	Question about whether before pregnancy the mother wanted to get pregnant then, later, or not at all or was unsure, respectively plus her initial happiness about becoming pregnant on a five point Likert scale of: very happy; somewhat happy; somewhat unhappy; very unhappy; and unsure. Postnatal 1–6 months	47%	SFGA	Age Education Ethnicity Smoking Previous LBW / PTB	OR 1.2 (0.6, 2.3)	No data but says non-significant relationship	Retrospective, recall, live births only. Sample only includes over 15, English and Spanish speaking women. Only those giving birth in Feb-May each year	No
Pulley et al. (2002), USA	1995 National Survey of Family Growth (NSFG) women with a single live birth in the last 5 years (4120)	Included all pregnancies reported in the 1995 NSFG ending in a live birth in the 5 years prior to the woman’s interview. The 1995 version of the intendedness questions was used and all women were asked how happy they were when they learned they were pregnant	NSFG question: plus a 10-point scale measuring happiness about a pregnancy. Postnatal mixed	31%	LBW <2500	Marital status Age Education Parity SEC Ethnicity	6% unwanted, 5.1% intended, says not sig	–	Retrospective, recall, live births only	Yes
Korenman et al. (2002), USA	Women in the National Longitudinal Survey of Youth (NLSY) who had at least one birth after 1978 (7800)	Uses data from the 1979–92 NLSY from women who had at least one birth after 1978	Four questions about pregnancy intention described as similar to those in the NSFG but not specified. Postnatal mixed	37%	LBW <2500	None	10.4% mother only intended, 7% if both intended	No data	Mostly focused on comparisons of intention between the partners. Uses models that attempt to account for uncontrolled confounding factors through within family comparisons on intended and unintended births	Yes
Ahluwalia et al. (2001), USA	13 states women with singleton births (15,219)	Analysis of PRAMS data for 13 states linked to birth certificates	PRAMS questions. Postnatal 1–6 months	45%	SFGA	None	OR 1.25 (0.99, 1.58)	–	Retrospective, recall, live births only. SFGA data only, not comparable	No
Eggleston et al. (2001), Ecuador	DHS (2490)	Subsample of the DHS	DHS questions. Postnatal >12 months		Women’s assessment of size and LBW <2500	ANC Smoking Alcohol consumption Age Parity rural v urban Education	OR 1.64 (1.22, 2.20)	Unwanted OR 1.64 (1.22, 2.20), mistimed OR1.18 (0.88, 1.60)	Uses mother’s assessment of size for outcome, as well as numeric answer if that was available. Had to exclude a large number of women who did not report their infant’s weight - these women may be more likely to have characteristics associated with LBW and therefore would lead to an underestimate	No
Joyce et al. (2000), USA	Women in cohort giving birth between 1979 and 1992 (7751)	National Longitudinal Survey of Labor Market Experience, a probability sample of young adults between the ages of 14 and 21 in 1979, the first year of the survey. Respondents are interviewed annually. Have sibling data so can compare intendedness within families	Four questions about pregnancy intention described as similar to those in the NSFG but not specified. Postnatal mixed	44%	LBW <2500	None	11.6% in unwanted, 6.4% in intended, no sig test	Only effect sizes presented so not able to compare	Multiple models with different control groups and confounders. Uses mother’s report of birthweight, says that there are high rates of agreement between this and vital records	Yes
Sable and Wilkinson (2000), USA	Missouri live singleton births between Dec 1 1989 and Mar 31 1991 (2378)	National Institute of Child Health and Human Development / Missouri Maternal and Infant Health Survey, a population based case control study. Cases were vLBW, matched with 2 controls next births—one LBW and one NBW, identified from birth certificate data	NSFG questions. Postnatal mixed	57.9%	LBW <2500 and vLBW	None	0R 0.90 (0.74, 1.10) for moderately LBW (1500–2499) v normal	–	Mixed methods of data collection and variable time to data collection. Retrospective, recall. Weights compared differently to other studies; vLBW v normal and moderate LBW v normal so not comparable	No
Colley et al. (1999), USA	Mothers who had a live-born infant in the 13 included states in 1997	Analysis of PRAMS data for 18 states linked to birth certificates	PRAMS questions. Postnatal 1–6 months	33–59%	LBW <2500	None	No data but says no differences in LBW by intention	–	Methods robust but some repeated data from other PRAMS studies in the same states and same year. Also no data presented to enable a calculation	No
Fourn et al. (1999), Benin	Women attending ANC in Cotonou hospital (4113)	Used a questionnaire at first ANC to gather socio-demographic data, obstetric history. Followed up through pregnancy and complications were recorded	Women were asked at the first visit if the pregnancy was wanted or unwanted. Antenatal	17%	LBW <2500	Pregnancy complications Age Parity	13% unwanted in NBW, 21.7% unwanted in LBW	OR 1.6 (1.30, 2.00)	Only women attending ANC at the hospital, but they say that about 90% of the local population access this. Not entirely clear what is adjusted for in the analysis	Yes
Kost et al. (1998), USA	NMHIS women aged 15–49 in 48 states who had a live birth in 1988 or NSFG nationally representative sample of women aged 15–44 who gave birth 1984–88 (11,585)	Data from national maternal and infant health survey and the national survey of family growth, NMHIS postal questionnaire, women aged 15–49 in 48 states who had a live birth in 1988. NSFG interviews with nationally representative sample of women aged 15–44 who gave birth between 1984 and 1988, birth certificates	NSFG questions. Postnatal mixed	n/a	LBW <2500 and LBW <10%	None	LBW more common in unwanted (9.7%) and mistimed (6.5%) pregnancies than intended pregnancies (5.1%), p < 0.05	-	Can’t calculate odds ratio as numeric data not presented. Lots of interesting data from the regression models. Importance of planning for early recognition of pregnancy and timely ANC	No
Mitchell and McCormack (1997), USA	Participants included women who had a live birth, a stillbirth, or an infant death in 1988 (18,000)	1988 NMIHS data and presentation of other analyses of the 1982–88 NSFG data. The women completed a 40-minute mailed questionnaire regarding prenatal care, health habits, and pregnancy outcomes	NSFG questions. Postnatal mixed	40%	LBW <2500	None	10.8% in unwanted, 6.7% in wanted, says sig	–	Retrospective, recall, live births only	Yes
Bitto et al. (1997), Chile, Italy, Columbia, USA	All women in the study who became pregnant between Jan1987 and Sept 1990 in the 5 centres (656)	Sub-study of a multicentre international prospective cohort study of women using natural family planning methods designed to ascertain the effects of timing of conception on pregnancy outcome. Intention determined on recognition of pregnancy, followed up at 16 and 32 weeks and after delivery	Women asked at time pregnancy recognised whether the pregnancy had been planned. Validated by natural family planning instructor after talking to woman and reviewing her chart and independently reviewed. Defined as planned if women stated it was her intention to become pregnant and her chart showed intercourse during her fertile period. Antenatal	51%	LBW <2500 and average birthweight	Age pregnancy complications BMI Smoking / alcohol Parity Previous LBW/PTB infant sex	OR 0.69 (0.30, 1.58)	OR 0.90 (0.24, 3.44)	Users of natural family planning - not representative of a wider population—Doesn’t present results by centre despite stating that they were very different—Chile had 37–40% unplanned pregnancies; DC had <2%—seems like very different populations, only have one OR for all data	No
Bustan and Coker (1994), USA	Pregnancies in women who were members of the Kaiser Health Plan in the East Bay Area of San Francisco (20,754)	Women recruited on confirmation of pregnancy, interviewed and data extracted from medical records	Wording of question not included but asked about the attitude of the woman and her partner to the pregnancy. Antenatal	14.4%	LBW <2500	None	Not presented and no raw data	OR 1.72 (1.08, 2.76)	Excluded unmarried women and those who said that their attitude to the pregnancy was different to their husbands. Excluded those with missing data on intention or who were not interviewed during pregnancy, and selected one pregnancy per women reduced the sample size to 8823 from over 20 000, also women who had access to this health care plan - suggests that these women were at less risk of UIP and of adverse outcomes raising issues of representativeness	No
Sharma et al. (1994), USA	High risk inner city women with a live birth between 1989 and 1991 in one of six geographical areas (1004)	Telephone interviews of 1004 women of childbearing ages (15 to 44 years), selected through random digit dialling procedure	Exact questions not presented but based on PRAMS and MIHS. Postnatal mixed	54%	LBW <2500	Age Marital status Education Ethnicity SEC maternal conditions Previous abortion	1.1 (0.9–1.4) according to paper, no raw data given	–	Selected high-risk populations, excludes those without telephones who are likely to be more disadvantaged. Live births only. Don’t present data, only adjusted OR and it isn’t clear what was included in the model	No
Gadow et al. (1998), Argentina (9), Bolivia (1), Brazil (4), Chile (1), Colombia (2) and Venezuela (1)	5155 normal women having normal offspring in 18 maternity hospitals participating in the ECLAMC in 1992–94 (5155)	As the control group of a south American study on malformations 5155 normal women having normal offspring were interviewed during the post-partum period in 18 maternity hospitals participating in the ECLAMC. This data is analysed here	(i) if this gestation was intended or not; and (ii) if contraceptive methods were used or not.. Postnatal <1 month	50%	Average birthweight	Age Education Parity Ethnicity	No differences in average birthweight, but raw data nor presented	–	Multi-centre study with results presented as aggregate may mask differences at country level. Retrospective but closer to delivery. Only live births. Slightly difference measure of intention. Only presents average birthweight	No
Poland et al. (1990), USA	200 poor, mainly black women who delivered at the Hutzel Hospital in Detroit over a 26 month period (200)	Interview and review of medical records	Initial attitude to pregnancy recorded as mixed, negative or positive. Time delay before telling second person about the pregnancy. Postnatal <1 month	50%	LBW <2500	None	Attitude to pregnancy contributes to variation in prenatal care which contributes to 26% of the variation in birthweight	–	Develops a model to assess the contribution of different factors to LBW. Doesn’t quantify difference in LBW between intention groups. Intention not robustly assessed and not comparable to others	No
Cartwright (1988), UK	Random sample of women with live births that year in 10 areas in England (1486)	Sub-study of a methodological study to assess the feasibility of monitoring maternity services through postal questionnaires. Random sample of births in each area (areas chosen systematically with a random starting point and with probability proportional to the number of births in 1982) in 1 month in 1984. Postal questionnaires sent about 4 months after birth with up to two reminders	‘When you first found you were pregnant, how did you feel about it then? Would you rather it had happened a bit later or were you pleased you were pregnant then, or sorry it had happened at all?’ ‘Around the time you became pregnant were you or your husband or partner generally using any method of birth control? ‘So would you say you intended to become pregnant or not? Stated intentions used in the analysis. Postnatal 1–6 months	27%	LBW <2500	None	Decreasing proportion of unintended pregnancies as birthweight increases	–	Retrospective, live births but comment on all conceptions. Measure of intention not standardised but similar to others	Yes
Marsiglio and Mott (1998), USA	6015 women in the NLSY who were interviewed in 1984 and who had had one child (1581)	Uses data from the 1979–92 National Longitudinal Survey of Youth (NLSY) from women who had at least one birth after 1978	‘Was the reason you (were not/stopped) using any contraceptive methods because you yourself wanted to become pregnant?’ Those who answered ‘no’ were then asked, ‘Just before you became pregnant did you want to become pregnant when you did?’ if ‘no’, ‘Did you want a baby but not at that time, or did you want none at all?’. Postnatal mixed	45%	LBW <2500	Age Ethnicity	7.9% in unwanted, 6.1% in wanted, says not sig	Can’t calculate but says not significant	Looking at direct and indirect relationships between intention and LBW through antenatal behaviours. Does multivariate regression	Yes
Pamuk and Mosher (1988), USA	Women aged 15–44 in the USA 7969	Data from the NFSG 1982 survey. Personal interviews were conducted with a multistage area probability sample of 7969 women 15–44 years of age	NSFG questions. Postnatal mixed	40%	LBW <2500	None	No data but says no differences noted in LBW by intention	–	Retrospective, live births only	No
McCormick et al. (1987), USA	Low income women in central Harlem (458)	Women attending for ANC at Harlem Hospital and affiliated clinics completed a questionnaire covering socio-demographics, attitude to child, health behaviours and exposure to stressful events	A series of questions on attitude to the pregnancy including ‘Did you plan this pregnancy?’ and whether they were surprised at being pregnant. Antenatal	73%	LBW <2500	None	No data but says no differences were noted in LBW by intention	–	No data presented so can’t calculate, says no relationship between planning and LBW / PTB. A group of low-income urban women, not many differences between planned and unplanned on socio-demographic characteristics or behaviours therefore no differences in outcomes not so surprising. Only presents data on women who attended for ANC	No
Laukaran and Van Den Berg (1980), USA	Ever married women who were a member of the Kaiser Foundation Health Plan in the San Francisco (12,391)	Data from the Child Health and Development studies, a prospective analysis of maternal attitude in relationship to events during pregnancy and delivery and pregnancy outcomes. Outcomes from medical records	How do you feel about having a baby now?’ 7 options divided into 4 categories of attitude and then pregnancy divided into wanted if there was a strong favourable response and unwanted if there was a negative response; Antenatal	28.20%	LBW <2500	None	No data but says no differences were noted in LBW by intention	–	Prospective study but measure of intention not comparable, only compares two extremes and middle section ignored. No data presented so can’t calculate - just says that no relationship between attitude and LBW	No
Morris et al. (1977), USA	Random selection of women giving birth in one of the hospitals included in the Family Planning evaluation (7921)	Interview on the postnatal ward of a random selection of women with live births in 60 hospitals in 17 major cities	Just before you became pregnant this time, did you: (1) want to become pregnant, (2) want a(nother) baby, but didn’t want to become pregnant yet, or (3) not want a(nother) baby? (Go to Q. 9b) (4) didn’t matter. 9b. At the time you became pregnant, did you feel (1) you would want a(nother) baby some time later (2) you would never want a(nother) baby?. Postnatal <1 month	22.1% in blacks, 10.9% in whites	LBW <2500	Stratified analyses by education, parity, marital status	6.6% in unwanted, 4.3% in wanted in whites (p < 0.05), 10.1% in unwanted, 9% unwanted in blacks, not sig	–	Retrospective, but very near to birth so less potential for recall bias, live births only	Yes

In brief, 27 of these studies were from HICs, two were from LICs (Ethiopia and Benin), two from lower-middle-income countries (LMICs) (one each from Ghana and Egypt), four from upper-middle-income countries (UMICs) (three from Iran and one from Ecuador) and two presented data from several countries.

To assess pregnancy intention, the USA studies tended to use either the National Survey of Family Growth or Pregnancy Risk Assessment Monitoring System questions; the studies in LICs were mostly based on the Demographic and Health Survey questions (see Appendix 1 for question wording). Questions about planning or wanting a pregnancy, attitude towards the pregnancy or about the timing of the pregnancy were all considered to be assessing pregnancy intention by these studies. However, these are different dimensions of the concept of pregnancy intention. Only seven had assessed intentions during pregnancy, the other 30 asked women any time from shortly after delivery to up to 5 years after the birth.

**Fig. 2:**
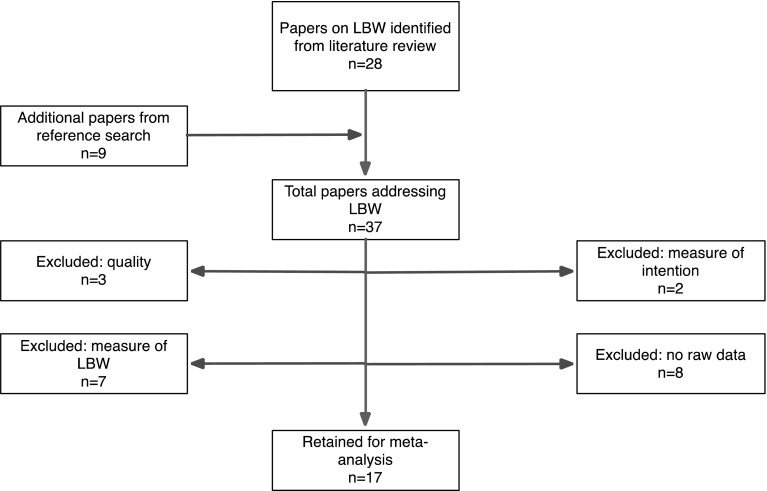
Flow chart of the studies in the pregnancy intention and LBW meta-analysis

On full-text review 20 studies were excluded, as shown in Fig. 2, leaving 17 for inclusion in the meta-analysis.

### Description of Included Studies

Of the 17 studies remaining, there were 14 from HICs, two from LICs and one from a LMIC (Ghana). Only two had assessed intentions during pregnancy and followed up women after birth (Fourn et al. 1999; Wado et al. 2014), the other 15 were retrospective, cross-sectional surveys. Study sample size ranged from just over 500–25,000 women as many were large, nationally representative surveys.

### Meta-Analyses of the Unadjusted Relationship

**Fig. 3:**
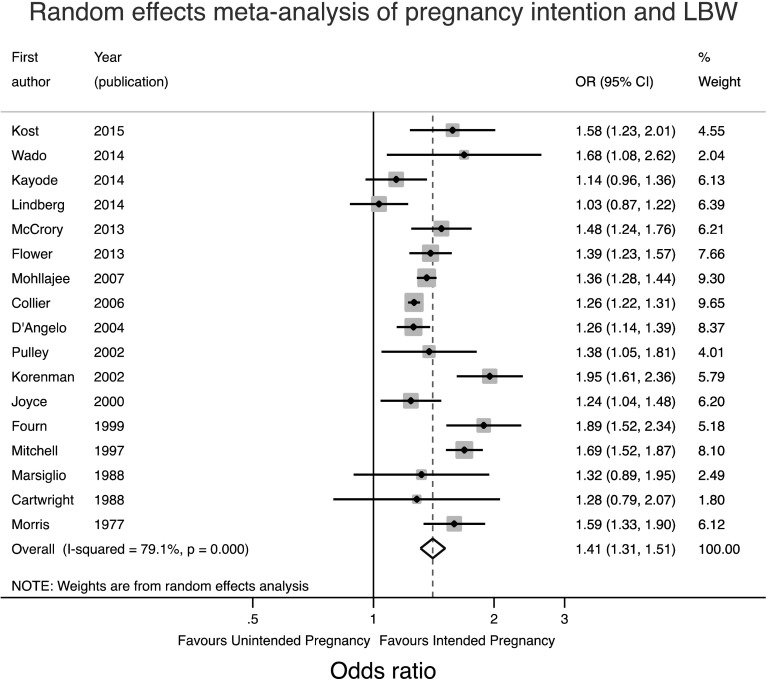
Forest plot of the random effects meta-analysis of studies assessing the relationship between pregnancy intention and LBW

Given the range of different confounders adjusted for in different studies, the raw data were first used to calculate unadjusted odds ratios for the meta-analysis, recognising that the apparent strength of association might be inflated by confounding. Given the significant and substantial heterogeneity between studies a random effects meta-analysis was performed as shown in Fig. 3.

**Fig. 4:**
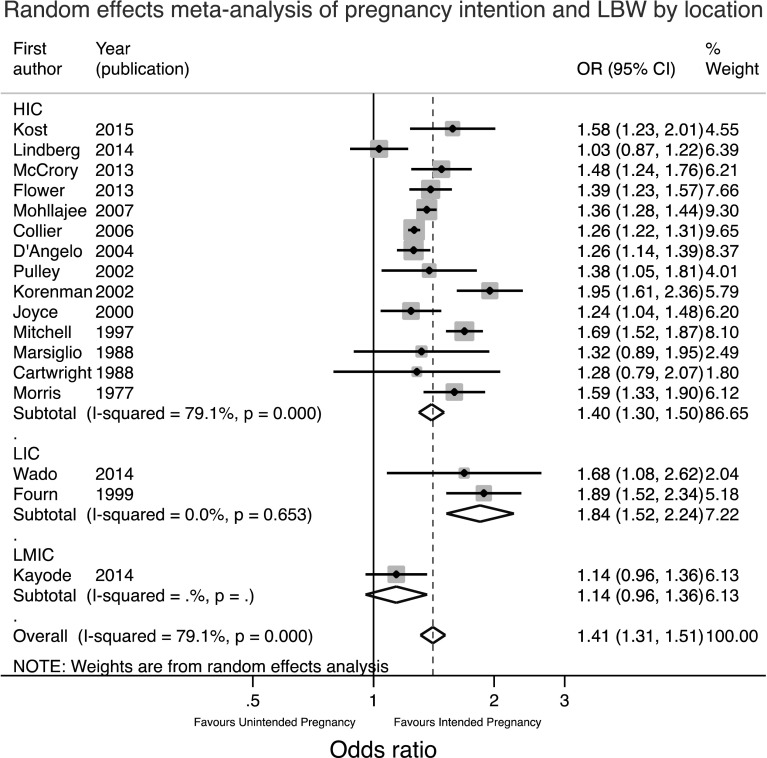
Forest plot of the random effects meta-analysis of studies assessing the relationship between pregnancy intention and LBW stratified by location

**Fig. 5:**
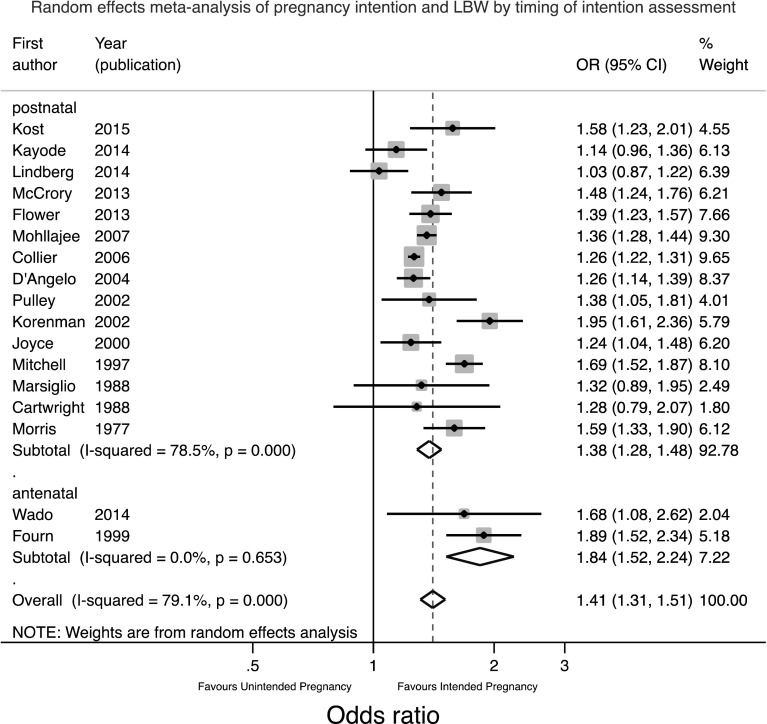
Forest plot of the random effects meta-analysis of studies assessing the relationship between pregnancy intention and LBW stratified by timing of assessment of intention

This meta-analysis suggests that the odds of having a LBW baby are 1.41 times greater in women who have an unintended pregnancy (95%CI 1.31, 1.51). The heterogeneity seen may be a result of the range of locations or timing or method of assessment of pregnancy intention. Therefore separate meta-analyses were conducted stratified for these factors and are shown in Figs. 4 and 5.

Figure 4 shows that the two LIC studies had a significantly higher combined OR of 1.84 (95%CI 1.52, 2.24) compared to 1.40 (95% CI 1.30, 1.50) in HIC countries. There was considerable heterogeneity between HIC countries. Figure 5 shows similar findings. Since the two antenatal studies were also the two studies in LICs it is not possible to say whether the higher pooled OR in these studies was due to the location or the timing of assessment. Theoretically speaking, the antenatal assessment of pregnancy intention should lead to a smaller effect size estimate as the potential for recall bias or for the outcome to influence the reported intention has been removed. On the other hand, the setting may lead to a larger effect size as the consequences of an unintended pregnancy may be more significant in a resource constrained environment.

### Findings of Adjusted Analyses

Out of these 17 studies, six calculated aORs. Two studies found non-significant relationships after adjustment (Lindberg et al. 2014; McCrory and McNally 2013). In two studies the findings remained significant with aORs of 1.60 (95%CI 1.30, 2.0) (Fourn et al. 1999) and 1.24 (95%CI 1.04, 1.48) (Flower et al. 2013). The final two studies had mixed findings. Wado et al. found that unwanted pregnancies remained significantly associated with LBW [aOR 2.08 (95%CI 1.02, 4.23)] when compared with intended pregnancies, but mistimed pregnancies did not (Wado et al. 2014). Mohllajee et al., however, found that neither unwanted nor mistimed pregnancies had a relationship with LBW after adjusting for confounders, but women who were ambivalent had increased odds of LBW [aOR 1.15 (95%CI 1.02, 1.29)] (Mohllajee et al. 2007). The fact that unintended pregnancies have been divided into different subcategories further complicates any comparison. Moreover, no two studies controlled for the same mix of confounders, which ranged from socio-demographic and obstetric history factors to smoking behaviour and uptake of antenatal care, which may be another explanation for these discrepancies.

### Publication Bias

**Fig. 6:**
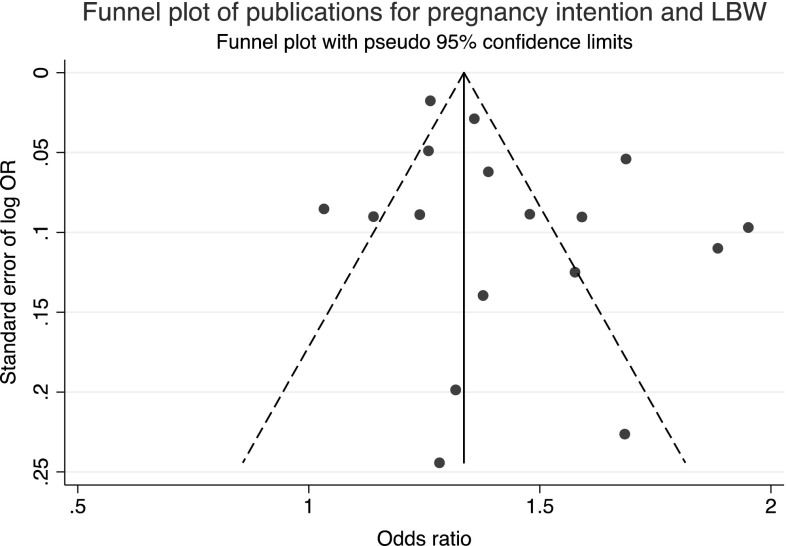
Funnel plot for pregnancy intention and LBW

The funnel plot to check for publication bias or small study effects is shown in Fig. 6. The lack of studies in the bottom left hand corner indicates that smaller studies with negative findings are missing. This may be a consequence of publication bias or that stronger effects are seen in smaller studies perhaps because of different methodology.

## Discussion

For LBW the meta-analyses of the unadjusted data suggest that unintended pregnancies (unwanted/mistimed/ambivalent combined) are associated with 1.41 times greater odds of having a low birthweight baby (95%CI 1.31, 1.51) than an intended pregnancy. This is in keeping with the findings of the previous meta-analysis. This finding seems robust to the location of the study and the timing of the assessment of pregnancy intention, though with the limited data available from either LICs or prospective studies this is not certain.

There are varying amounts of data for the relationships between pregnancy intention and pregnancy outcomes. There is a suggestion that pregnancy loss may be higher in unintended pregnancies in LICs and that unintended pregnancies are associated with increased neonatal mortality in both HICs and LICs after adjusting for confounders. However, there are limited data in this area and there is no exploration of the mechanism of effect, whether through biological studies, psychosocial stress or uptake of services.

Despite much academic debate around the construct and measurement of pregnancy intention, this does not appear to have translated into methodologically improved research in this area; the methodological limitations highlighted by Tsui et al. (2010) persist. Studies continue to be dominated by cross-sectional, retrospective surveys where pregnancies are dichotomised into intended or unintended on the basis of a single question. The various questions used, whether asking about timing, desire or happiness, are all assumed to be measuring the same construct, despite evidence to the contrary (Trussell et al. 1999). Some studies have begun to disaggregate unintended pregnancies into mistimed and unwanted during analysis and in doing so are uncovering differential effects and determinants, reinforcing the need for a more refined measure of pregnancy intention (Mohllajee et al. 2007; Shah et al. 2011; Wado et al. 2014). To date very few studies have assessed pregnancy intention using a psychometrically validated measure, and none of the LBW studies in this review had, which could be one reason for the inconsistencies seen between studies.

Furthermore, the effect of pregnancy intention on pregnancy outcomes is likely to be confounded or mediated by a number of other factors. The determinants of pregnancy intention are often the same as the risk factors for adverse outcomes. For instance, a fourth or subsequent pregnancy is both more likely to be unintended (Bustan and Coker 1994; Flower et al. 2013; Mohllajee et al. 2007) and to have an adverse outcome (Bai et al. 2002). In addition, pregnancy intention and some of its determinants are related to lower uptake of preventative care practices during the antenatal, intra-partum and postnatal periods and to higher levels of risky behaviours during pregnancy, which are also known to increase the risk of adverse outcomes (Lindberg et al. 2014; Marston and Cleland 2003; Mohllajee et al. 2007; Shaheen et al. 2007). However, most studies have not sufficiently accounted for this in their analyses. There is some suggestion from adjusted analyses of papers included in this review that confounders or mediators, such as socio-economic status, smoking (in HICs), maternal nutrition and uptake of antenatal care, may explain the effect of pregnancy intention on increased risk of LBW (McCrory and McNally 2013; Mohllajee et al. 2007; Wado et al. 2014).

## Limitations

There are two main limitations to this review. Firstly, the searches were only conducted on databases and therefore did not include unpublished studies, the grey literature or consultation with experts. This could mean that relevant studies were missed; nevertheless we identified more studies than previous reviews in these areas had. Shah et al. (2011), for example, only identified ten studies on low birth weight. Secondly, we did not contact authors to obtain raw data if it had not been presented in the study. This meant that some eligible studies could not be included in the analysis and seemed to be more likely to occur when studies found no relationship. This could lead to an over-estimate of the relationships between pregnancy intentions and outcomes. Furthermore we did not seek to obtain individual participant data that would have allowed a more detailed and coherent meta-analysis.

## Conclusion

This review has highlighted persistent gaps and flaws with the existing evidence. The general lack of studies in developing countries noted by Gipson et al. (2008) persists, though there have been more studies in these areas over the last few years. These studies have tended to find a greater risk of adverse outcomes with unintended pregnancy.

Current evidence suggests that there may be a relationship between pregnancy intention and pregnancy outcome. To confirm this, and to understand how this relationship is mediated, longitudinal studies are required. Pregnancy intention should be measured before birth and data on the potential confounders and mediators, including maternal background characteristics, pre-conception, antenatal, delivery and postnatal behaviours, should be collected. A psychometrically valid measure of pregnancy intention that assesses intention on a continuous scale, such as the London Measure of Unplanned Pregnancy (Barrett et al. 2004), should be used in preference to dichotomous measures and the full range of scores should be used in the analysis. A better understanding of the way in which pregnancy intention influences pregnancy outcome will enable us to tailor pre-conception, antenatal, delivery and postnatal services to meet women’s needs and reduce the risk of adverse outcomes. While these methodological advances are required in research in high-income countries, research in low-income countries, where arguably the consequences of unintended pregnancies are much greater, is urgently needed.

## Appendix

See Table 4.

**Table 4:** Wording of questions to assess pregnancy intention

Survey name	Pregnancy intention questions on survey
National Survey of Family Growth (NSFG) from 1973, 1976, 1982, 1988, 1995, 2002, 2006–10 and 2011–13. USA	1. Was the reason you (were not/stopped) using any method because you, yourself, wanted to become pregnant? (Yes/No - If yes, go to Q4, If no, go to Q2) 2. At the time you became pregnant, did you, yourself, actually want to have a(nother) baby at some time? (Yes/No/Don’t know - If yes, go to Q4, If no, go to Q5, if don’t know, go to Q3) 3. It is sometimes difficult to recall these things, but, as you look back to just before that pregnancy began, would you say you probably wanted a(nother) baby at some time or probably not? (If probably yes, go to Q4, if probably no or didn’t care, go to Q5) 4. Did you become pregnant sooner than you wanted, later than you wanted, or at about the right time? (Sooner/ later/right time/didn’t care) 5. And what about your partner at the time you became pregnant... did he want you to have a(nother) baby at some time? (Yes/no/don’t know - If yes, go to Q6) 6. Did you become pregnant sooner than he wanted, later than he wanted, or at about the right time? (Sooner/later/ right time/didn’t care) Additional questions used in 1995 survey (For all women) Which number on the card best describes how you felt when you found out you were pregnant? (Card has a 10-point scale from 1—very unhappy to 10—very happy) (For women under 25) Which number on the card best describes your opinion about becoming pregnant? (Card has a 10-point scale from 1 strongly disagree to 10—strongly agree) You were worried that you did not know enough about how to take care of a baby You thought that a new baby would keep you from doing the things that you were used to doing like working, going to school, going out and so on You looked forward to teaching and caring for a new baby You looked forward to the new experiences that having a baby would bring You looked forward to experiencing the changes in your body that come with carrying a baby You looked forward to telling your friends that you were pregnant You were worried about what being pregnant would do to your body You were worried that you did not have enough money to take care of a baby You dreaded telling your friends that you were pregnant You looked forward to buying things for a new baby
Pregnancy Risk Assessment Monitoring System (PRAMS) from 1987 to date. USA	1. Thinking back to just before you were pregnant, how did you feel about becoming pregnant? a. I wanted to be pregnant sooner b. I wanted to be pregnant then c. I wanted to be pregnant later d. I didn’t want to be pregnant then or at any time in the future
Demographic and Health Survey	At the time you became pregnant did you want to become pregnant then, did you want to wait until later, or did you want to have no (more) children at all?

## Footnotes

1 They defined perinatal mortality as deaths from 4 months of pregnancy to 28 days after birth.
